# Thermal damage width and hemostatic effect of bipolar electrocoagulation, LigaSure, and Ultracision techniques on goat mesenteric vessels and optimal power for bipolar electrocoagulation

**DOI:** 10.1186/s12893-019-0615-4

**Published:** 2019-10-22

**Authors:** Jun Liang, Huimin Xing, Yali Chang

**Affiliations:** 1 Department of Obstetrics and Gynecology, The Third Hospital of Hebei Medical University, 139 Ziqiang Road, Shijiazhuang, 050051 Hebei Province People’s Republic of China; 2 Department of Obstetrics and Gynecology, Shijiazhuang Maternal and Child Health Hospital, Shijiazhuang, 050082 Hebei Province China

**Keywords:** Bipolar electrocoagulation, Advanced bipolar, Ultrasonic, Thermal damage, Hemostatic effect

## Abstract

**Background:**

We aimed to determine the optimal bipolar electrocoagulation power for laparoscopic surgery and to investigate which method, bipolar electrocoagulation, advanced bipolar, or ultrasonic technique was more reliable.

**Methods:**

Goat mesenteric vessels (210 in vivo samples) with diameters of 3.03–5.44 mm were selected. Bipolar electrocoagulation with 80 W, 75 W, 70 W, 65 W, 60 W, 55 W, and 50 W, and advanced bipolar and ultrasonic techniques were performed on mesenteric vessels. The thermal damage width, hemostatic effect, and burst pressure of these tissues were recorded. SPSS version 13.0 was used for all data analysis.

**Results:**

The results showed that 60 W was the optimal for bipolar electrocoagulation based on the thermal damage width, hemostatic effect, and burst pressure. In contrast, the thermal damage width of advanced bipolar and ultrasonic techniques was smaller than that of bipolar electrocoagulation, and advanced bipolartechnique had the highest successful rate for hemostasis and highest burst pressure.

**Conclusions:**

Bipolar electrocoagulation was optimally performed with 60 W of power. Compared with ultrasonic and bipolar electrocoagulation techniques, advanced bipolar use was more reliable for mesenteric vessels in laparoscopic surgery; however, bipolar electrocoagulation with optimal power can be used for its simplicity of operation and low cost.

## Background

Laparoscopic surgery is widely used in gynecological surgery. Hemostasis is a fundamental principle of surgery. Owing to the quest for more efficient and safer hemostatic techniques in laparoscopic surgery, several coagulating techniques and devices have been developed, including bipolar electrocoagulation, advanced bipolar (like LigaSure) and ultrasonic device (such as Ultracision) [1].

LBipolar electrocoagulation is still widely used in laparoscopic surgery because of its simplicity of operation and low cost [2, 3]. Several studies suggested that LigaSure and plasma knife devices caused only minor thermal damage and had better hemostatic effects [4–6]. LHowever, some surgeons are unaware of the thermal damage caused by bipolar electrocoagulation and use of LigaSure, and Ultracision devices. Lack of skilled operation can result in vessel bleeding at the site of coagulation during and after surgery, leading to ureter, bladder, and bowel damage. The incidence rate of ureteral injury is 0.3–3.8% [7]. Importantly, such damage is difficult to localize during surgery, and symptoms such as ureteral leaks usually present 7–10 days after surgery, adversely affecting both the surgeon and patient [8, 9]. Therefore, it is necessary to determine the optimal power for bipolar electrocoagulation, and to study the thermal damage and hemostatic effects of bipolar electrocoagulation, LigaSure, and Ultracision techniques.

The present study investigated thermal damage to mesenteric vessels and hemostatic effects in goats using bipolar electrocoagulation at different power levels. The thermal damage and hemostatic effects of bipolar electrocoagulation, advanced bipolar (LigaSure) and ultrasonic device (Ultracision) were compared. We aimed to determine suitable bipolar electrocoagulation power for use in laparoscopic surgery, as well as to investigate which method, bipolar electrocoagulation, advanced bipolar, or ultrasonic technique, was more reliable.

## Methods

### Materials

**Fig. 1:**
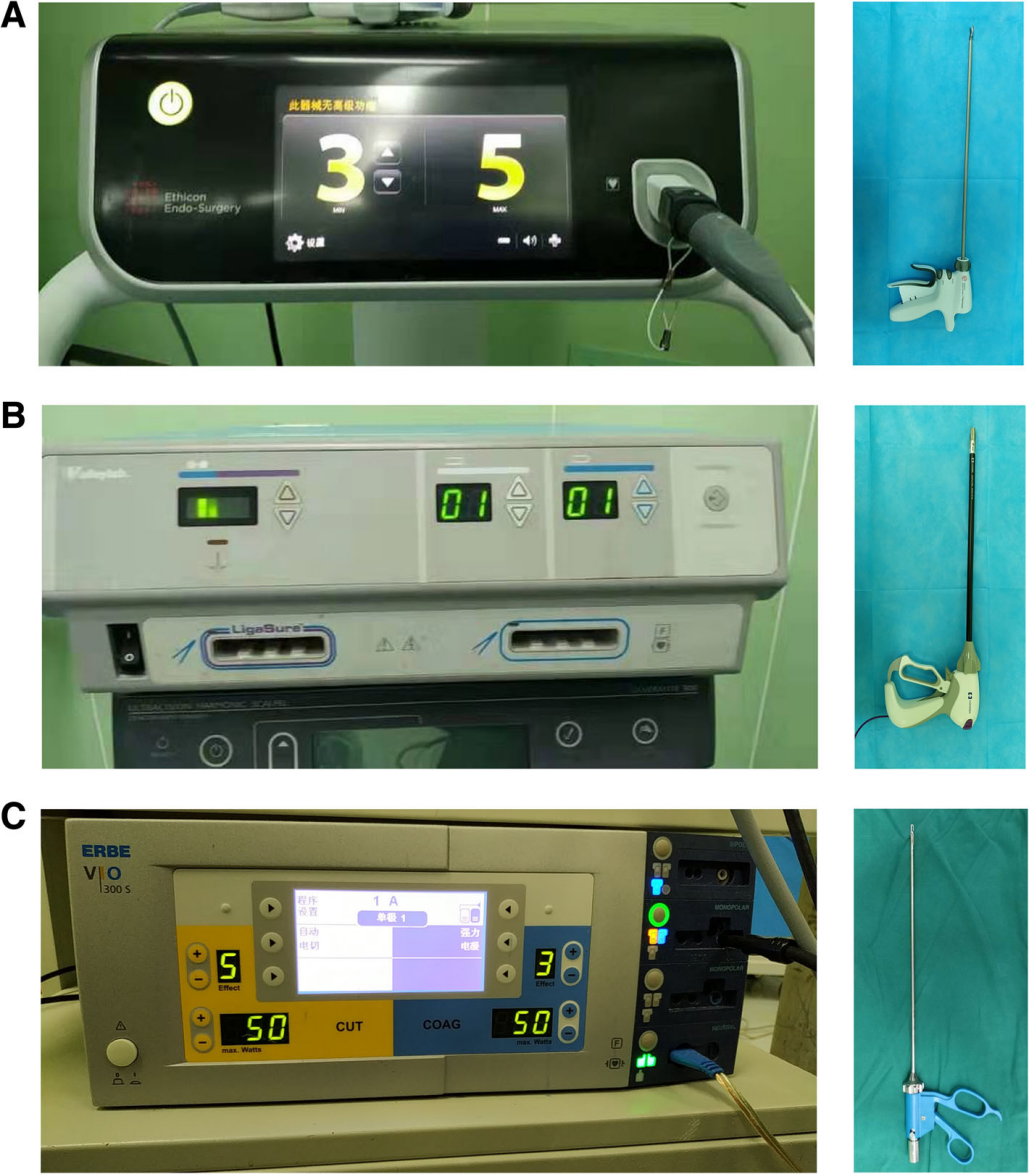
The devices of Ultracision (**a**) LigaSure (**b**), and bipolar electrocoagulation (**c**)

Twelve healthy adult goats (with good mental state and normal diet, and without illness presentation) weighing 35–41 kg were obtained from the animal experimental center of Hebei Medical University. Mesenteric vessels with diameters of 3.03–5.44 mm were selected using Vernier calipers, and 210 in vivo samples were used for bipolar electrocoagulation (30 cases each, using 80 W, 75 W, 70 W, 65 W, 60 W, 55 W, and 50 W); in addition, 80 samples were treated using the LigaSure device and 80 were treated using the Ultracision device. The bipolar electrocoagulation can provide high-frequency electric energy for tissues through the two clamps of the bipolar forceps to dehydrate and solidify the tissue so as to stop bleeding. The working principle of Ultracision is to convert electrical energy into mechanical energy through a special conversion device. After high-frequency ultrasonic vibration, the water in the tissue cells is vaporized, the protein hydrogen bond is broken, and the tissue is cut after solidification. The figures for three devices are shown in Fig. 1. A 20.00 mm × 20.00 mm section with tissue thickness of 5.00 mm was excised from the position of forceps holder (KINGE 5 × 330 mm straight head screw using bipolar electrocoagulation forceps, forceps-blade 15 × 4 mm). Tissue was embedded, sectioned, and stained with hematoxylin and eosin. After preparation, the tissue was observed under microscopy.

The study was approved by the Medical Ethic Committee of the Third Hospital of Hebei Medical University.

### Methods

The goats were fixed on an operating table in supine position, and intravenous anesthesia was performed with 35 mg/kg ketamine, continuously administered as needed. A midline abdominal incision was made and mesenteric vessels with a diameter of 3.03–5.44 mm were selected. Vessels were divided using LigaSure, Ultracision, and bipolar electrocoagulation techniques, and the cut ends, together with 20 mm × 20 mm tissue sections, were excised. A high-frequency electrosurgical system (ERBE VIO300S, Germany) was set to electrocoagulation mode, and tissues were treated using different power levels (80 W, 75 W, 70 W, 65 W, 60 W, 55 W, and 50 W). LigaSure was set at 3/5, and Ultracision was set at 3. For the bipolar coagulation ‘Bipolar Soft Coag’ was used. If the hemostatic effect was inadequate, electrocoagulation was repeated. Operations were performed by the same person, using consistent operative technique. Goats were sacrificed after surgery with 30 mg kg-1 of 5% solution of sodium pentobarbital (Sigma, St. Louis, MO, USA).

### Criteria for successful hemostasis

Electrocoagulation with bipolar forceps (5 mm in diameter; Hangzhou Kangji Medical Instruments Co., China) was performed until no thermal bubbling was generated, or until the sensor system automatically terminated operation with use of LigaSure (with 5 mm (diameter) vessel closure forceps; Valleylab, American) and Ultracision (with 5 mm (diameter) tool head; Johnson & Johnson, American) devices. Absence of bleeding after incision was considered successful electrocoagulation. If bleeding persisted, the need for repeated electrocoagulation was considered failure of a single electrocoagulation treatment.

### Observation and measurement

Using an optical microscope equipped with a standard micromeasurement instrument, and with the lateral margin of the electrocoagulation forceps as the starting point, measurement of thermal damage distance was made by the same pathologist in all cases. The width of thermal damage was the vertical distance from the region with pathological changes of thermal damage to the broken ends. The recording unit was mm, and the measurement was accurate to 0.01 mm.

Burst pressure was also measured. First, a catheter was inserted into the open end of the vessel segment and secured. Second, normal saline was infused into the arterial lumen at a fixed rate (Lambda VIT-FIT, LAMBDA Laboratory Instruments, Zurich, Switzerland), and the pressure was recorded using a pressure transducer (Greißinger Electronic GMH3150, Regenstauf, Germany). Then, the maximum pressure (in mmHg) before leakage at the sealing site was defined as burst pressure. All burst pressure measurements were performed by two researchers, blinded for the respective study groups.

### Statistical analysis

SPSS version 13.0 (SPSS, Chicago, IL, USA) was used for all data analysis. The chi-square test was used to compare the hemostatic effect (success rate of single vessel closure) of single electrocoagulation treatments at various specific power levels (50 W, 55 w, 60 W, and 65 W), and *P* < 0.05 was considered a significant difference. The Kruskal-Wallis test was used to analyze the diameter of mesenteric vessels divided with the 3 different devices. One-way analysis of variance was used for comparison of thermal damage width, *P* < 0.05 was considered a significant difference, and the least significant difference test was used for pairwise comparison.

## Results

### Pathological changes in thermally damaged tissue

**Fig. 2:**
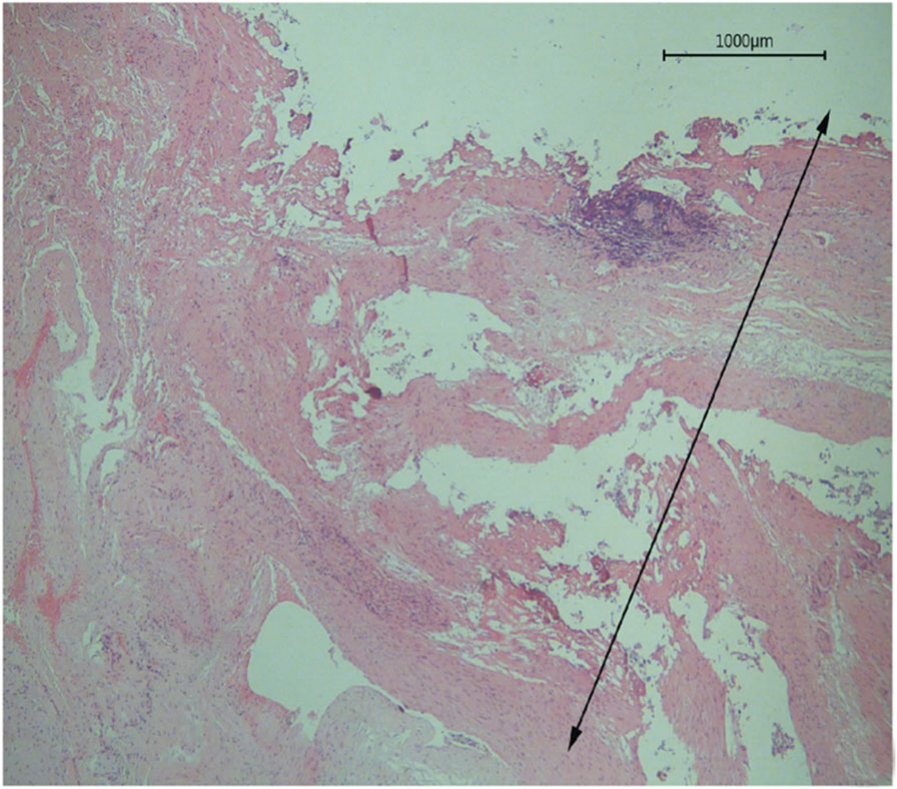
Pathological changes in thermally damaged tissue. (× 100; arrow represents thermal damage width)

The mesenteric vessels showed acute coagulation necrosis. The outer layer of thermal damage showed carbonization or solidification, and the carbonization zone was relatively thin, with loss of normal cell morphology. The solidification zone was located in the carbonization zone, with a number of tissue cell layers arranged closely and oriented in one direction, showing cell elongation, cell membrane damage, patchy cytoplasm, vacuole formation, and eosinophilic enhancement. The nuclei were ruptured and the nuclear material was condensed, dissolved, and fragmented. Chromatin was blurred and indistinct, or fused with cytoplasm, without epithelial stripping, and epithelial tissue remained intact (Fig. 2).

### Comparison of goat mesenteric vascular diameters

Goat mesenteric vessels (3.03–5.44 mm in diameter) with diameter comparable to human uterine arteries [3] were selected. The mesenteric vessel diameter for each group is shown in Table 1. There was no significant difference in the diameter of blood vessels in each group (χ^2^ = 0.901, *P* = 0.999).

**Table 1:** The mesenteric vascular diameter, thermal damage width and burst pressure for bipolar electrocoagulation under different powers, LigaSure and ultracision

Group	Cases	Mesenteric vascular diameter (mm)	Thermal damage width (mm)	Burst pressure (mmHg)
80 W	30	4.194 ± 0.755	7.967 ± 0.350	660.267 ± 38.596
75 W	30	4.230 ± 0.672	7.889 ± 0.359	645.200 ± 36.096
70 W	30	4.231 ± 0.653	6.381 ± 0.406	637.000 ± 42.559
65 W	30	4.228 ± 0.673	6.301 ± 0.378	609.867 ± 30.854
60 W	30	4.311 ± 0.612	5.091 ± 0.332	553.567 ± 37.058
55 W	30	4.188 ± 0.543	5.013 ± 0.320	546.567 ± 34.834
50 W	30	4.204 ± 0.701	6.256 ± 0.339	518.533 ± 25.322
LigaSure	80	4.211 ± 0.678	4.470 ± 0.693	1033.575 ± 82.025
Ultracision	80	4.256 ± 0.605	4.089 ± 0.762	673.538 ± 50.454

### Comparison of thermal damage width of mesenteric vessels

There were statistically significant differences in thermal damage width between vessels treated at 75 W and 80 W in comparison with other wattages (*P* < 0.001), and the thermal damage width of mesenteric vessels was maximal at 75 W and 80 W. However, there was no statistically significant difference between treatment at 75 W and 80 W (*P* = 1.000). There were statistically significant differences in thermal damage width between vessels treated at 55 W and 60 W in comparison with other wattages (*P* < 0.001), and the thermal damage width of mesenteric vessels was minimal at 55 W and 60 W with bipolar electrocoagulation. However, there was no significant difference between treatments at 55 W and 60 W (*P* = 1.000). In addition, the difference was not statistically significant at 50 W compared with 65 W and 70 W (*P* = 1.000), and thermal damage width was similar for these 3 groups (Table 2).

**Table 2:** Comparison of the thermal damage width of the mesenteric vascular tissue with bipolar electrocoagulation under different powers

Group	Cases (mm)	*P* value						
		1	2	3	4	5	6	7
80 W	7.967 ± 0.350	–	1.000	< 0.001	< 0.001	< 0.001	< 0.001	< 0.001
75 W	7.889 ± 0.359	1.000	–	< 0.001	< 0.001	< 0.001	< 0.001	< 0.001
70 W	6.381 ± 0.406	< 0.001	< 0.001	–	1.000	< 0.001	< 0.001	1.000
65 W	6.301 ± 0.378	< 0.001	< 0.001	1.000	–	< 0.001	< 0.001	1.000
60 W	5.091 ± 0.332	< 0.001	< 0.001	< 0.001	< 0.001	–	1.000	< 0.001
55 W	5.013 ± 0.320	< 0.001	< 0.001	< 0.001	< 0.001	1.000	–	< 0.001
50 W	6.256 ± 0.339	< 0.001	< 0.001	1.000	1.000	< 0.001	< 0.001	–

χ^2^ = 182.161, *P* < 0.001

The thermal damage width of mesenteric vessels using different electric devices was as follows: bipolar electrocoagulation group (5.091 ± 0.332 mm) > LigaSure group (4.470 ± 0.693) > Ultracision group (4.089 ± 0.762); (χ^2^ = 40.430, *P* < 0.001 for pairwise comparison).

### Comparison of the hemostatic effect of single bipolar electrocoagulation treatments at several specific powers (65, 60, 55, and 50 W) and using different electric devices

The hemostatic effect of single bipolar electrocoagulation treatments at different powers and using different electric devices is shown in Table 3. The success rate of vessel closure after single electrocoagulation treatments at 70 W, 75 W, and 80 W was 100%, but the thermal damage width was large. Thus, we compared the hemostatic effect of single electric coagulation treatments at several lower powers (65, 60, 55, and 50 W), and there were statistically significant differences among these four groups (*P* < 0.05). Significant differences were observed between 60, 55, and 50 W (*P* < 0.05), and the success rate was higher at 60 W. No statistically significant difference was observed in the success rate for vessel closure after single electrocoagulation treatments at 65 W and 60 W, but there were statistically significant differences in thermal damage width between 65 W and 60 W (*P* < 0.05). Thus, 60 W was the optimal power for bipolar electrocoagulation because of the lower thermal damage width and better hemostatic effect.

**Table 3:** The hemostatic effect for single bipolar electrocoagulation under different powers and for different electric appliance

Groups	Cases	Number of closed vessel after single electric coagulation	Successful rate of vascular closure after single electric coagulation
80 W	30	30	100.00%
75 W	30	30	100.00%
70 W	30	30	100.00%
65 W	30	28	93.33%
60 W	30	26	86.67%
55 W	30	18	60.00%
50 W	30	16	53.33%
LigaSure	80	80	100.00%
Ultracision	80	73	91.25%

In addition, LigaSure had the highest single success rate for hemostasis and showed significant differences with Ultracision and bipolar electrocoagulation (*P* < 0.05). No significant difference was found between Ultracision and bipolar electrocoagulation (*P* > 0.05) techniques.

### Comparison of burst pressures

The comparison of burst pressure for bipolar electrocoagulation at different powers is shown in Table 4. No statistically significant difference was found for pairwise comparison of 80 W, 75 W, 70 W, and 65 W (*P* > 0.05), and these four groups had higher burst pressures. Furthermore, there was no significant difference for pairwise comparisons of 60 W, 55 W, and 50 W (*P* > 0.05), and these three groups had lower burst pressures. Significant differences were found between 80 W, 75 W, 70 W, 65 W, and 60 W, 55 W, 50 W (χ^2^ = 147.204, *P* < 0.001).

**Table 4:** Comparison of burst pressure for bipolar electrocoagulation under different powers

Group	burst pressure (mmHg)	*P* value						
		1	2	3	4	5	6	7
80 W	660.267 ± 38.596	–	1.000	1.000	0.057	< 0.001	< 0.001	< 0.001
75 W	645.200 ± 36.096	1.000	–	1.000	0.548	< 0.001	< 0.001	< 0.001
70 W	637.000 ± 42.559	1.000	1.000	–	1.000	< 0.001	< 0.001	< 0.001
65 W	609.867 ± 30.854	0.057	0.548	1.000	–	0.004	0.001	< 0.001
60 W	553.567 ± 37.058	< 0.001	< 0.001	< 0.001	0.004	–	1.000	0.935
55 W	546.567 ± 34.834	< 0.001	< 0.001	< 0.001	0.001	1.000	–	1.000
50 W	518.533 ± 25.322	< 0.001	< 0.001	< 0.001	< 0.001	0.935	1.000	–

χ^2^ = 147.204, *P* < 0.001

In addition, the comparison of burst pressure for bipolar electrocoagulation, LigaSure, and Ultracision techniques was as follows: LigaSure (1033.575 ± 82.025 mmHg) > Ultracision (673.538 ± 50.454) > bipolar electrocoagulation (553.567 ± 37.058) (χ^2^ = 158.205, *P* < 0.001 for pairwise comparison).

## Discussion

Laparoscopic surgery is widely used in gynecologic surgery because of less trauma, faster recovery, shorter hospital stay, less postoperative pain, and less scarring. Bipolar electrocoagulation, advanced bipolar device, and ultrasonic device are commonly used coagulation devices. We studied the thermal damage to mesenteric vessels and hemostatic effects in goats using bipolar electrocoagulation at different powers, and the thermal damage and hemostatic effect of bipolar electrocoagulation, advanced bipolar device, and ultrasonic device were compared. The results showed that 60 W was a suitable power for bipolar electrocoagulation. Furthermore, the thermal damage width of advanced bipolar device, and ultrasonic device was smaller than that of bipolar electrocoagulation, and bipolar device had the highest single success rate of hemostasis and highest burst pressure.

Ying et al. [10] reported a bilateral uterine blood vessel diameter of 4.731 ± 0.658 mm in 200 healthy fertile woman using color Doppler, and there was no significant difference between the left and right sides. The infundibulopelvic ligament was also similar in diameter. Thus, goat mesenteric vessels, with diameters close to those of human uterine vessels and the infundibulopelvic ligament, were selected for our research. In the present study, the power for bipolar electrocoagulation was set at 50–80 W, comparable to the range of electrocoagulation power used in laparoscopic total hysterectomy. The lower the power between 55 and 80 W, the less the thermal damage width. The difference was not statistically significant for 50 W compared with 65 W and 70 W (*P* = 1.000), because the success rate of vessel closure after single electrocoagulation treatment decreased (53.33%) after power reduction. Furthermore, repeated electrocoagulation led to heat accumulation, and the range of thermal damage was bound to increase.

The success rate of vessel closure after single bipolar electrocoagulation treatment was 100.00% with power between 70 W and 80 W, suggesting that if the blood vessel was fully exposed and the uterine vessel or ovarian suspensory ligament was not in contact with the ureter, it was appropriate to choose high power. However, high power will lead to an increase in secondary injury because of thermal damage. Therefore, we studied the hemostatic effect of single bipolar electrocoagulation at several specific powers (65, 60, 55, and 50 W). Significant differences were observed between 60 W and 55 W as well as 50 W (*P* < 0.05), and the success rate was higher at 60 W. No statistically significant difference was observed for the success rate of vessel closure after single electric coagulation treatment between 65 W and 60 W (*P* < 0.05), but there was statistically significant difference for thermal damage width between 65 W and 60 W (*P* > 0.05). Furthermore, no statistically significant difference was observed for burst pressure between 60 W and 65 W. Thus, 60 W was a suitable power for bipolar electrocoagulation, based on the thermal damage width, hemostatic effect, and burst pressure. In laparoscopic hysterectomy, the power of bipolar electrocoagulation can be adjusted on the basis of 60 W to achieve an optimal hemostatic effect. In addition, the success rate of vessel closure after single bipolar electrocoagulation was not 100.00% at 60 W; thus, suturing and ligation of vessel ends is safe and reliable after dividing uterine vessels with electrocoagulation.

In the present study, the thermal damage width of the mesenteric vessels using different electric devices was as follows: bipolar electrocoagulation group > advanced bipolar device group > ultrasonic device group. This was consistent with the study by Diamantis et al. [1]. With advances in laparoscopic technique, various new electrosurgical instruments will cause less damage, making surgery more accurate and minimally invasive. Although the thermal damage width of bipolar electrocoagulation is large, it is still reasonably safe [3]. If the infundibulopelvic ligament and uterine vessels can be fully exposed, it is appropriate to use bipolar electrocoagulation. However, when the anatomical level is not clear because of adhesions, LigaSure or Ultracision can be chosen after lysis of adhesions to reduce secondary ureteral injuries [11], especially with Ultracision, which has a lower operating temperature compared with LigaSure and bipolar electrocoagulation, and can cause tissue protein coagulation and degeneration forming a coagulation block at 50–100 °C.

Advanced bipolar device had the highest single-treatment success rate for hemostasis and showed a significant difference compared with ultrasonic device and bipolar electrocoagulation (*P* < 0.05). No significant difference was found between ultrasonic device and bipolar electrocoagulation (*P* > 0.05). The findings indicated that advanced bipolar device was the safest way to close a vessel, and the hemostatic effect was equal to that of a vascular clamp and suture line [12]. In addition, the burst pressure was as follows: advanced bipolar device > ultrasonic device > bipolar electrocoagulation (χ^2^ = 158.205, *P* < 0.001 for pairwise comparisons). The vascular coagulation effect of advanced bipolar device is clear, but the repeated use of an advanced bipolar device knife head causes dulling and the possibility of poor results. Therefore, surgeons should not blindly trust devices, and should individualize hemostatic techniques based on operative conditions.

There are several limitations in this study. First, testing alternative modes and generators were not done. Additionally, this study is an animal experiment, and we will deepen the study to investigate the differences of thermal injuries of more different electrosurgical instruments. Further clinical studies are still needed.

## Conclusions

In conclusion, 60 W was an optimal power setting for bipolar electrocoagulation based on the thermal damage width, hemostatic effect, and burst pressure. Bipolar electrocoagulation had the largest thermal damage width of the mesenteric vessels, advanced bipolar device the second largest, and ultrasonic device the least. The advanced bipolar technique had the highest single success rate of hemostasis compared with ultrasonic device and bipolar electrocoagulation. Furthermore, the burst pressure of mesenteric vessels among all three techniques varied as advanced bipolar > ultrasonic device > bipolar electrocoagulation. Thus, compared with ultrasonic device and bipolar electrocoagulation techniques, advanced bipolar device is more reliable for mesenteric vessels in laparoscopic surgery when considering first attempt sealing alone without regard to cost; however, bipolar electrocoagulation with optimal power can be used because of its simple operation and low cost; when considering both cost and first attempt sealing, a higher energy setting is required with bipolar electrocoagulation.

## Data Availability

The datasets used and/or analysed during the current study available from the corresponding author on reasonable request.
